# Google Trends Predicts Present and Future Plague Cases During the Plague Outbreak in Madagascar: Infodemiological Study

**DOI:** 10.2196/13142

**Published:** 2019-03-08

**Authors:** Nicola Luigi Bragazzi, Naim Mahroum

**Affiliations:** 1 Department of Health Sciences Postgraduate School of Public Health University of Genoa Genoa Italy; 2 Sackler Faculty of Medicine Tel-Aviv University Tel Aviv Israel

**Keywords:** plague, infodemiology, infoveillance, infectious outbreaks, Google Trends, nowcasting and forecasting models, digital surveillance

## Abstract

**Background:**

Plague is a highly infectious zoonotic disease caused by the bacillus *Yersinia pestis*. Three major forms of the disease are known: bubonic, septicemic, and pneumonic plague. Though highly related to the past, plague still represents a global public health concern. Cases of plague continue to be reported worldwide. In recent months, pneumonic plague cases have been reported in Madagascar. However, despite such a long-standing and rich history, it is rather difficult to get a comprehensive overview of the general situation. Within the framework of electronic health (eHealth), in which people increasingly search the internet looking for health-related material, new information and communication technologies could enable researchers to get a wealth of data, which could complement traditional surveillance of infectious diseases.

**Objective:**

In this study, we aimed to assess public reaction regarding the recent plague outbreak in Madagascar by quantitatively characterizing the public’s interest.

**Methods:**

We captured public interest using Google Trends (GT) and correlated it to epidemiological real-world data in terms of incidence rate and spread pattern.

**Results:**

Statistically significant positive correlations were found between GT search data and confirmed (*R*^2^=0.549), suspected (*R*^2^=0.265), and probable (*R*^2^=0.518) cases. From a geospatial standpoint, plague-related GT queries were concentrated in Toamasina (100%), Toliara (68%), and Antananarivo (65%). Concerning the forecasting models, the 1-day lag model was selected as the best regression model.

**Conclusions:**

An earlier digital Web search reaction could potentially contribute to better management of outbreaks, for example, by designing ad hoc interventions that could contain the infection both locally and at the international level, reducing its spread.

## Introduction

Plague and history have always been strongly interrelated since the earliest description of plague pandemic [1]. Excluding the so-called *plague of Athens*, which could have been caused by typhus or other microorganisms, the first well-authenticated mention of plague dates back to the 6th century: the *Justinian Plague*, named after the Byzantine emperor. This outbreak took place in Egypt in 542 AD and spread across the Mediterranean basin regions, killing more than 25 million people [2]. Three large pandemics occurred afterward, including a major outbreak during the decline of the Eastern Roman Empire, the rapid weakening of the Persian Empire, and the subsequent tumultuous rise of the Islamic Empire [3]. Another major episode was the Great Plague Pandemic, which began in China in 1333-1334 during a period of famine and spread to Europe by trade causing millions of deaths [4]. The third major outbreak was the Modern Plague Pandemic, which originally occurred in China in 1860, spreading worldwide by rats on trade ships leading to over 10 million deaths across the world [5,6].

This highly infectious zoonotic disease is caused by the bacillus *Yersinia pestis*, a member of the *Enterobacteriaceae* family [7]. Transmission of plague occurs when infected rodents’ fleas bite humans. Human-to-human transmission is possible when transmitted by infected air droplets. Three major forms of the disease are known: (1) bubonic plague, the most common form of plague, which is characterized by acute febrile illness accompanied by enlarged and tender lymph nodes; (2) septicemic plague, characterized by sepsis manifested by fever and systemic illness, generally without preceding symptoms; and (3) pneumonic plague, which may be primary when acquired directly by infected air droplets and secondary when it spreads to the lung from other infected sites of the body—both of these forms lead to a highly contagious and lethal disease [8].

Diagnosis of plague is made based on the above-mentioned clinical findings accompanied with suspected history of exposure. Identification by culture as well as increased paired serum titers are suitable diagnostic methods. Plague can be fatal if left untreated. Besides supportive therapy for severely ill patients, treatment is composed of systemic antibiotics of aminoglycoside-based therapy.

Though highly associated with the past, plague still represents a global public health concern. Cases of plague continue to be reported worldwide, especially in Africa but also in Asia, South America, and even in the United States. According to the World Health Organization (WHO), 3248 cases of plague were reported from 2010 until 2015, with 584 deaths [9]. Recently, foci have been described in Libya and Algeria [8]. The highest incidence of plague cases in recent years is reported from Madagascar. In fact, cases of bubonic plague are reported annually in Madagascar since the first case was introduced in 1898; however, recent reports show a large outbreak of pneumonic plague occurring in major urban cities, which is different from what was previously reported (ie, cases concentrated mainly in rural areas). In recent months, we have witnessed the spread of plague to the Seychelles islands, reflecting a further escalation of the current outbreak [10-12].

However, despite such a long-standing and rich history, it is rather difficult to get a comprehensive overview of the general situation. Within the framework of electronic health (eHealth), in which people surf the internet more and more looking for health-related material, new information and communication technologies, such as Web 2.0, portable computers, mobile phones and devices, as well as social media and social networks, could enable researchers to get a wealth of data, which could complement traditional surveillance of infectious diseases.

In this study, we aimed to assess public reaction regarding the recent plague outbreak in Madagascar by quantitatively characterizing this interest and correlating it to epidemiological *real-world* data, in terms of incidence rate and spread pattern.

## Methods

Google Trends (GT) is a free open-source tool used to track and observe internet search activity [13]. GT was used to assess recent search activity with regard to the recent plague outbreak in Madagascar. To that end, GT was mined from August 1 to November 17, 2017 [13]. This particular time frame was chosen in order to better capture the temporal dynamics of the plague outbreak, monitoring the internet-related activity before (ie, digital behavior at the baseline) and during the epidemic. During the drafting and production of this study, the latest available WHO situation report, released on November 17, 2017 [14], was utilized.

The GT search tool has two options for searching keywords: searches can be performed by *search term* or by *search topic*. While the former enables the user to search for exact keywords, the latter option uses a broader search that finds all Web searches containing the inserted keyword(s) or related terms.

The results given by GT are output as normalized values (ie, relative search volumes [RSVs]) rather than absolute, raw values. Every query is divided by the total searches performed in a given geographic region and time range and normalized to a scale between 0 and 100 based on the topic’s popularity in comparison to all searches carried out in that region and time frame. For further details concerning GT, the reader is referred to Nuti et al’s review of GT and its potential applications in the medical field [15]; the reader is also referred to Mavragani et al’s recent systematic review concerning methods, tools, and statistical approaches and techniques in the field of GT research [16].

In this study, the second search option (ie, searching by topic with related terms) was used. Specifically, we searched for “Plague (Topic).” Searches were geographically limited to Madagascar. In Madagascar, the language spoken is Malagasy, while the second official language is French. English is spoken by less than 20% of the population. It is widely known that the sample when using online queries cannot be representative; however, in our case, searching for “Plague” and selecting the *search topic* option enabled us to overcome any linguistic issue related to the diffusion of the language. This approach ensured the robustness of our results.

Correlational analysis and multivariate regression models for nowcasting and forecasting, with lags up to 7 days, were performed based on the GT results with the number of confirmed, suspected, and probable cases of plague as reported by the WHO situation report. Different regression models were run, computing the different fitting parameters, including *R*^2^ and adjusted *R*^2^, and the best model was chosen according to the Akaike Information Criterion (AIC) values.

Statistical analyses were performed with the commercial software XLSTAT 2017 (Addinsoft). All values with *P* values less than .05 were considered statistically significant.

## Results

**Figure 1:**
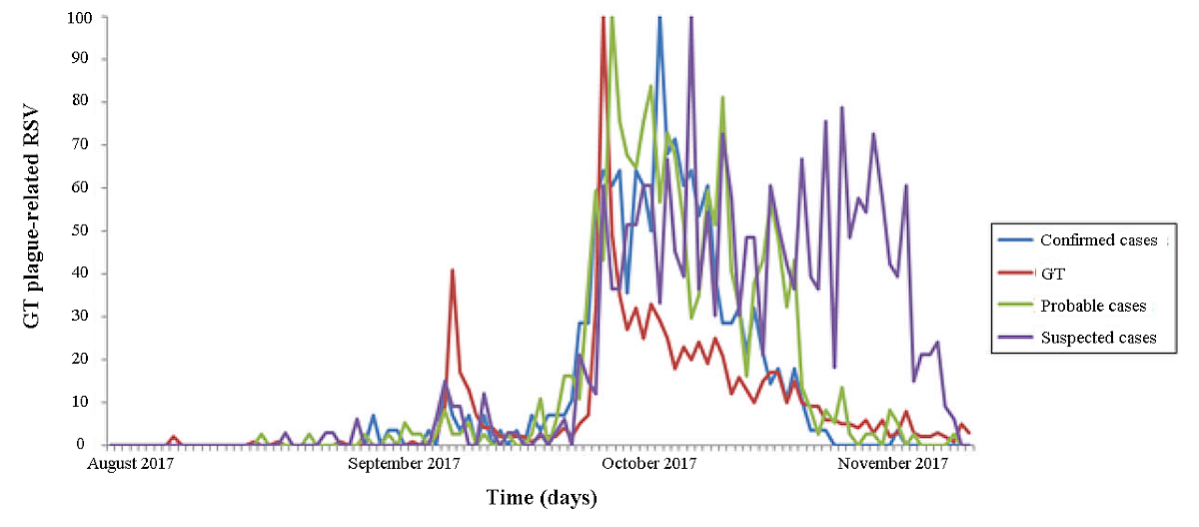
Time trends of plague cases (confirmed, probable, and suspected) and plague-related Google Trends (GT)–generated data. All data are normalized for comparison purposes. RSV: relative search volume.

Average plague-related search activities, expressed as RSVs, are shown superimposed on the trends of new suspected, probable, and confirmed cases of plague in Figure 1. The searches showed a small burst of activity on September 14, 2017, immediately after the official notification (ie, September 13, 2017) sent to the WHO by the Madagascar Ministry of Public Health of an outbreak of pneumonic plague in Madagascar. This notification followed the death of a young man some days before on September 11, 2017, who suffered from severe respiratory disease confirmed to be caused by plague. A very large spike was noticed during the first week of October 2017. Afterward, RSVs tended to decrease over time to slightly above baseline levels. Similarly, the incidence of suspected, probable, and confirmed cases of plague in Madagascar also exhibited a small spike in the third week of September 2017, and many more cases were confirmed during the recent outbreak starting in the first week of October.

**Figure 2:**
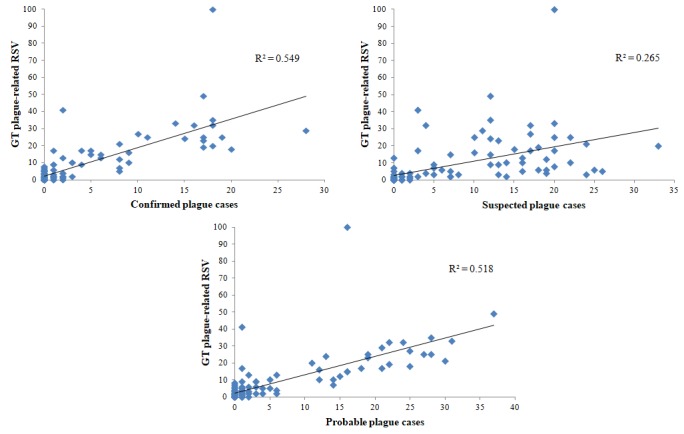
Scatterplots of the correlations between epidemiological values and the Google Trends (GT)–generated data related to the recent plague outbreak in Madagascar. RSV: relative search volume.

**Figure 3:**
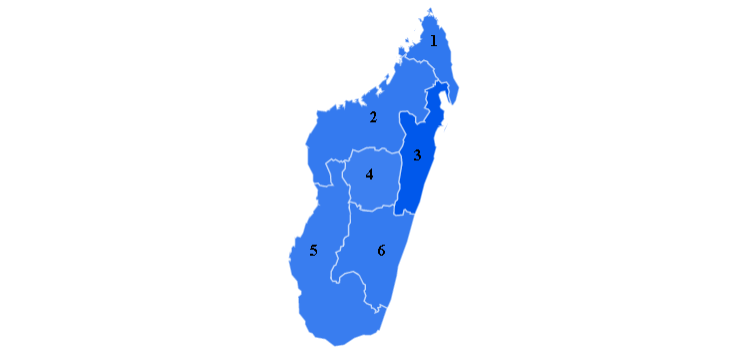
Spatial heat map of Google Trends–based plague-related data. The figure is based on the map provided by Google Trends. 1: Antsiranana Province; 2: Mahajanga Province; 3: Toamasina Province; 4: Antananarivo Province; 5: Toliara Province; and 6: Fianarantsoa Province. Color gradient correlates with the volume of plague-related Web searches.

The best nowcasting model in terms of AIC values (see Multimedia Appendix 1) showed that new confirmed cases of plague had a statistically significant association with GT-based RSVs (*P*<.001, beta coefficient 1.158), as shown in the multivariate regression analyses in Multimedia Appendix 2. Scatterplots of incident cases showed similar and statistically significant positive correlations with GT search data (*R*^2^=0.549, *P*=.001 for the confirmed cases; *R*^2^=0.265, *P*=.005 for the suspected cases; and *R*^2^=0.518, *P*=.001 for the probable cases; see Figure 2). From a geospatial standpoint, plague-related GT queries were concentrated in certain regions of Madagascar, most notably in Toamasina (100%), Toliara (68%), and Antananarivo (65%). A heat map of search density in different regions of Madagascar is shown in Figure 3. Concerning the forecasting models, the 1-day lag model was selected for regression analysis due to optimal AIC values (see Multimedia Appendix 3). This forecasting model shows that we can predict new probable cases up to 1 day in advance with statistically significant certainty (*P*<.001; see Multimedia Appendix 4).

## Discussion

### Principal Findings

Plague outbreak in Madagascar has drawn wide public attention shown here by our findings based on large Web search activity data analysis. Madagascar has been known as an endemic area of plague in its bubonic form with annually reported cases from April to September, generally across rural areas. However, the recent outbreak is characterized by pneumonic plague occurring in larger and more crowded cities. The current outbreak, known to be a highly contagious form of plague and in combination with the recent spread to the Seychelles islands, has its own distinctive features.

Monitoring and analyzing Web search activity manifested by novel data streams (NDS), especially during outbreaks, is of great importance in terms of surveillance as shown by O'Shea [17] in a recent systematic review. Big data or *vast digital data* analysis is, indeed, an opportunity to improve surveillance and epidemic intelligence, being inexpensive, transparent, and flexible. As such, *event-based internet biosurveillance* can act as an extension of traditional surveillance and monitoring systems and can be utilized as an additional data source, contributing, therefore, to a more comprehensive estimate of infectious diseases.

GT, based on Google search, is a freely accessed website tool, which provides data on how often a specific search item (ie, plague) is searched relative to total search volume worldwide or in specific areas and in different languages. For instance, in 2009, during the peanut butter-associated outbreak of *Salmonella enterica* subtype *Typhimurium*, GT provided preliminary evidence of an emerging infectious outbreak, enabling early disease detection [18]. Other research studies based on GT have shown the possibility of monitoring and tracking flu epidemics [19-21], as well as other infections [22-35].

In our study, bursts of searches of plague-related topics corresponded both spatially and temporally with the outbreak’s spatiotemporal trends across the region studied (ie, Madagascar). The role of Web-based NDS for outbreak surveillance is crucial for workers in the field of public health and safety. Plague-related digital behavior as captured by GT analysis reflected rapid public response to the pneumonic plague outbreak in Madagascar, with some minor search peaks occurring even before the formal declaration by the WHO. Moreover, this reaction seemed to decline rapidly afterward, whereas the WHO continued to release the report of additional confirmed plague cases.

In our study, it is interesting to note that the potential influence of prior awareness of a clinical case of plague, which occurred on September 13, 2017, on search behaviors of a population was reflected by the rapid increase of searches found on September 14, 2017. From September 30, 2017, people were probably more able to recognize specific signs and symptoms related to plague due to news or public campaigns. In this case, the suspicion of disease may lead people to seek confirmatory Web information, contributing to the increase of the activity of internet users. These arguments could be used to explain the highest value of *R*^2^ when including confirmed, probable, and suspected cases in multivariate regression models (ie, searches were probably motivated or driven by personal impressions and knowledge of disease).

Findings from the regression analyses showed the feasibility of exploiting NDS for predicting (ie, nowcasting and forecasting) plague cases. Extant predictive models of plague are usually built within the *ecological-niche modeling framework*, in which geographic, environmental, and ecological parameters, such as landscape-scale environmental features, are utilized [36,37]. To the best of our knowledge, this is the first model incorporating plague-related information-seeking behavior in terms of Web-based NDS, such as GT. Even though a correlation between epidemiological values and Web searches could appear trivial, this is surprising, especially considering the poor internet penetration in Madagascar (ie, only 4%-5% of the population have access to the internet).

Despite its novelties, which are among the major strengths of the current investigation, our study suffers from some limitations, which should be properly recognized. The shortcomings include the fact that GT provides relative and not absolute values, thus hindering the possibility of further refining and processing them. Moreover, GT captures only Web searches carried out with the Google search engine, which is, on the other hand, the most utilized search tool. Another drawback was the relatively low values of *R*^2^. The limited internet penetration (ie, approximately 4%-5% of the entire population) as well as the short time frame chosen for the study could be among the factors explaining such values.

### Conclusions

Our study has shown an increase in digital Web searches with a unique pattern induced by the recent outbreak of plague in Madagascar. GT plays a highly important role in outbreak tracking and monitoring, in that it can capture public reaction and interest toward infectious disorders in real time before cases are formally communicated by the WHO. This earlier digital Web search reaction could potentially contribute to better management of outbreaks, for example, by designing ad hoc interventions that could contain the infection both locally and at the international level, reducing its spread.

## Appendix

Multimedia Appendix 1 Regression analyses for the nowcasting models.

Multimedia Appendix 2 Fitting parameters of the nowcasting models.

Multimedia Appendix 3 Regression analysis of the best forecasting model.

Multimedia Appendix 4 Fitting parameters of the forecasting models.

## Abbreviations

AIC: Akaike Information Criterion
eHealth: electronic health
GT: Google Trends
NDS: novel data streams
RSV: relative search volume
WHO: World Health Organization
