# Supplementary material for: Google Trends Predicts Present and Future Plague Cases During the Plague Outbreak in Madagascar: Infodemiological Study
Source: JMIR Public Health Surveill. 2019 Mar 8;5(1):e13142. doi: 10.2196/13142 (PMC6429048; doi:10.2196/13142)
Supplement: Multimedia Appendix 1 [file publichealth_v5i1e13142_app1.pdf]

Multimedia Appendix 1. Regression analyses for the nowcasting models.

Source	Value	Standard error	T	Pr > |t|	Lower bound (95%)	Upper bound (95%)	Standardized value	Standard error	Lower bound (95%)	Upper bound (95%)
**Confirmed cases**										
Intercept	0.271	1.731	0.157	0.876	-3.161	3.704				
Confirmed cases	1.611	0.145	11.140	**< 0.0001**	1.325	1.898	0.730	0.066	0.600	0.860
Time	0.041	0.028	1.444	0.152	-0.015	0.097	0.095	0.066	-0.035	0.224
**Probable cases**										
Intercept	0.993	1.844	0.538	0.591	-2.663	4.649				
Propbable cases	1.047	0.107	9.814	**< 0.0001**	0.836	1.259	0.700	0.071	0.559	0.842
Time	0.025	0.031	0.825	0.411	-0.036	0.087	0.059	0.071	-0.083	0.200
**Suspected cases**										
Intercept	4.132	2.343	1.763	0.081	-0.514	8.778				
Suspected cases	0.940	0.182	5.162	**< 0.0001**	0.579	1.300	0.576	0.112	0.355	0.797
Time	-0.039	0.048	-0.813	0.418	-0.135	0.056	-0.091	0.112	-0.312	0.130
**Confirmed + probable + suspected cases**										
Intercept	1.007	1.793	0.561	0.576	-2.549	4.562				
Confirmed cases	1.158	0.280	4.127	**< 0.0001**	0.601	1.714	0.524	0.127	0.272	0.776
Propbable cases	0.292	0.199	1.470	0.145	-0.102	0.687	0.195	0.133	-0.068	0.459
Suspected cases	0.175	0.162	1.079	0.283	-0.147	0.497	0.107	0.099	-0.090	0.304
Time	0.006	0.037	0.160	0.873	-0.067	0.078	0.014	0.085	-0.154	0.181
