# Supplementary material for: Google Trends Predicts Present and Future Plague Cases During the Plague Outbreak in Madagascar: Infodemiological Study
Source: JMIR Public Health Surveill. 2019 Mar 8;5(1):e13142. doi: 10.2196/13142 (PMC6429048; doi:10.2196/13142)
Supplement: Multimedia Appendix 2 [file publichealth_v5i1e13142_app2.pdf]

Multimedia Appendix 2. Fitting parameters of the nowcasting models.

Fitting parameters	Time factor only model	Confirmed cases model	Confirmed cases + time factor model	Probable cases model	Probable cases + time factor model	Suspected cases model	Suspected cases + time factor model	Confirmed + probable + suspected model	Confirmed + probable + suspected + time factor model
R^2	0.086	0.571	0.579	0.518	0.521	0.265	0.270	0.596	0.596
Adjusted R^2	0.078	0.567	0.571	0.514	0.512	0.258	0.256	0.584	0.580
MSE	172.760	81.148	80.333	91.092	91.365	138.919	139.361	77.886	78.615
RMSE	13.144	9.008	8.963	9.544	9.559	11.786	11.805	8.825	8.867
MAPE	196.130	65.872	67.746	59.600	62.629	111.917	102.208	48.800	49.282
DW	0.572	1.291	1.307	1.537	1.528	0.771	0.808	1.343	1.346
Cp	2.000	2.000	3.000	2.000	3.000	2.000	3.000	4.000	5.000
AIC	563.539	481.176	481.051	493.776	495.078	539.775	541.098	478.646	480.619
SBC	568.922	486.559	489.125	499.158	503.152	545.158	549.172	489.412	494.076
PC	0.948	0.445	0.445	0.500	0.506	0.762	0.771	0.435	0.443
