# Supplementary material for: Google Trends Predicts Present and Future Plague Cases During the Plague Outbreak in Madagascar: Infodemiological Study
Source: JMIR Public Health Surveill. 2019 Mar 8;5(1):e13142. doi: 10.2196/13142 (PMC6429048; doi:10.2196/13142)
Supplement: Multimedia Appendix 3 [file publichealth_v5i1e13142_app3.pdf]

Multimedia Appendix 3. Regression analysis of the best forecasting model.

Source	Value	Standard error	T	Pr > |t|	Lower bound (95%)	Upper bound (95%)	Standardized value	Standard error	Lower bound (95%)	Upper bound (95%)
**Confirmed + probable + suspected cases (forecasting – 1-day lag)**										
Intercept	0.615	1.723	0.357	0.722	-2.803	4.032				
Propbable cases	0.973	0.187	5.194	**< 0.0001**	0.601	1.344	0.649	0.125	0.401	0.897
Suspected cases	0.056	0.153	0.366	0.715	-0.247	0.359	0.034	0.093	-0.151	0.220
Time	0.013	0.035	0.378	0.706	-0.056	0.082	0.030	0.080	-0.128	0.188
Confirmed cases	0.302	0.264	1.141	0.256	-0.222	0.825	0.136	0.120	-0.101	0.373
