# Supplementary material for: Google Trends Predicts Present and Future Plague Cases During the Plague Outbreak in Madagascar: Infodemiological Study
Source: JMIR Public Health Surveill. 2019 Mar 8;5(1):e13142. doi: 10.2196/13142 (PMC6429048; doi:10.2196/13142)
Supplement: Multimedia Appendix 4 [file publichealth_v5i1e13142_app4.pdf]

Multimedia Appendix 4. Fitting parameters of the forecasting models.

**Fitting parameters**	**1-day lag**	**2-day lag**	**3-day lag**	**4-day lag**	**5-day lag**	**6-day lag**	**1-week lag**
R^2	0.645	0.565	0.476	0,479	0.460	0.419	0.477
Adjusted R^2	0.631	0.548	0.455	0,458	0.438	0.395	0.456
MSE	69.731	86.142	104.490	104,659	109.399	118.678	107.618
RMSE	8.350	9.281	10.222	10,230	10.459	10.894	10.374
MAPE	64.337	83.743	86.593	111,835	118.050	141.627	172.402
DW	1.217	1.273	1.206	1,046	1.030	1.031	0.760
Cp	5.000	5.000	5.000	5,000	5.000	5.000	5.000
AIC	463.302	481.671	497.682	493,201	493.156	496.845	482.090
SBC	476.712	495.035	510.999	506,471	506.378	510.019	495.214
PC	0.390	0.478	0.576	0,573	0.594	0.640	0.576
